# Antiadipogenic Effects of *Aster glehni* Extract: In Vivo and In Vitro Effects

**DOI:** 10.1155/2013/859624

**Published:** 2013-06-20

**Authors:** Heon-Myung Lee, Gabsik Yang, Tae-Gue Ahn, Myung-Dong Kim, Agung Nugroho, Hee-Juhn Park, Kyung-Tae Lee, Wansu Park, Hyo-Jin An

**Affiliations:** ^1^Department of Pharmacology, College of Oriental Medicine, Sangji University, Wonju-si, Gangwon-do 220-702, Republic of Korea; ^2^Department of Physiology, College of Oriental Medicine, Sangji University, Wonju-si, Gangwon-do 220-702, Republic of Korea; ^3^Department of Pharmaceutical Engineering, Sangji University, Wonju-si, Gangwon-do 220-702, Republic of Korea; ^4^Department of Pharmaceutical Biochemistry, College of Pharmacy, Kyung Hee University, Seoul 130-701, Republic of Korea; ^5^College of Oriental Medicine, Kyungwon University, Seongnam 461-701, Republic of Korea

## Abstract

*Aster glehni* (AG) is a Korean traditional herb that grows in Ulleungdo Island, Republic of Korea. None of the several reports on AG include a determination of the effect of AG on adipogenesis. The primary aim of this study was to determine whether AG attenuates adipogenesis in mouse 3T3-L1 cells and epididymal fat tissue. AG blocked the differentiation of 3T3-L1 preadipocytes in a concentration-dependent manner and suppressed the expression of adipogenesis-related genes such as *PPAR**γ***, *C/EBP**α***, and *SREBP1c*, the master regulators of adipogenesis. Male C57BL/6J mice were divided randomly and equally into 4 diet groups: control diet (CON), high-fat diet (HFD), HFD with 1% AG extract added (AG1), and HFD with 5% AG extract added (AG5). The experimental animals were fed HFD and the 2 combinations for 10 weeks. Mice fed HFD with AG gained less body weight and visceral fat-pad weight than did the mice fed HFD alone. Moreover, AG inhibited the expression of important adipogenic genes such as *PPAR**γ***, *C/EBP**α***, *SREBP1c*, *LXR*, and leptin in the epididymal adipose tissue of the mice treated with AG1 and AG5. These findings indicate antiadipogenic and antiobesity effects of AG and suggest its therapeutic potential in obesity and obesity-related diseases.

## 1. Introduction

Overweight and obesity are defined as abnormal or excessive fat accumulation that may impair health; globally, excessive body weight is the fifth leading risk factor for death. At least 2.8 million adults die each year as a result of obesity. Furthermore, 44% of the diabetes burden, 23% of the ischemic heart disease burden, and 7% to 41% of the burden of specific cancers are attributable to obesity [1]. Obesity is now a worldwide health problem regardless of age, sex, or ethnicity and is associated with fatal diseases [2]. 

The process by which undifferentiated preadipocytes become adipocytes is termed adipogenesis. The differentiation to adipocyte is a complex developmental procedure that involves the coordinated interplay of numerous transcription factors [3]. Peroxisome proliferator-activated receptor *γ* (PPAR*γ*) and CCAAT/enhancer binding proteins (C/EBP*α*), which are crucial transcription factors in adipogenesis, activate the expression of other adipocyte markers [4]. Sterol regulatory element binding protein 1c (SREBP1c) is also important in the regulation of obesity and adipocyte differentiation [5]. Lipoprotein lipase (LPL) is a transcription gene of the lipogenic enzyme [6], and liver X receptor (LXR) activation enhances lipogenesis [7]; leptin is a hormone secreted by adipose tissues that plays a key role in regulating energy intake and energy expenditure, including appetite/hunger and metabolism [8].

The common side effects of conventional pharmacological obesity treatments—hypertension, cardiac arrhythmia, constipation, headache, steatorrhea, and deficiencies of lipid-soluble vitamins and essential fatty acids [9, 10]—have contributed to a focus on herbal medicine as a healthcare modality. Several natural products extracted from herbal plants have been shown to be effective for specific conditions. Some natural extracts, as well as the principal components of the extracts, such as berberine and resveratrol, have antiobesity effects [11, 12]. Studies on the efficacy of herbal medicines are actively going on in all parts of the world.

*Aster glehni* Franchet et Sckmidt (AG), which is common to Ulleungdo Island, Republic of Korea, is officially recognized as a regional specialty that grows only in this region. It has recently been named *Aster pseudoglehni *Lim, Hyun & Shin. In the republic of Korea, the leaves of AG are used in a dish called chwinamul, and AG has also been used to treat diabetes mellitus, hypercholesterolemia, insomnia, and cardiovascular disease [13]. According to a recent study, AG extract has both sedative and anticonvulsant effects [14]. Previously, we reported that the AG rich in CQ compound lowers the total lipid, triglyceride, phospholipid, and cholesterol levels in animal model [15]. Although AG's effect on body weight in rats has been reported [15], no study has focused on the antiadipogenic effect of AG in vitro. Specific adipogenic signal genes were examined to elucidate the specific mechanism underlying the effect of AG. The present study considered the antiobesity and antiadipogenic effects of AG in 3T3-L1 preadipocytes and mice.

## 2. Materials and Methods

### 2.1. Plant Material and Preparation of the Extract

AG leaves from Ulleungdo Island, Republic of Korea, were dried and crushed for extraction. The dried material was refluxed with 70% EtOH for 6 h at 60°C. The extract was filtered, concentrated under reduced pressure, and then freeze-dried to obtain a solid extract powder.

### 2.2. Cell Culture

The 3T3-L1 mouse preadipocytes were cultured in Dulbecco's Modified Eagle's Medium (DMEM, Life Technologies Inc., Grand Island, NY, USA) supplemented with 10% bovine calf serum, 100 *μ*g/mL streptomycin, and 100 units/mL penicillin at 37°C in 10% CO_2_. The cells were grown to confluency in 10% calf serum/DMEM. Two days after the cells had reached confluency (day 0), they were stimulated with a methylisobutylxanthine, dexamethasone, and insulin (MDI) induction medium (DMEM containing 10% fetal bovine serum (FBS), 0.5 mM 3-isobutyl-1-methylxanthine (IBMX), 1 *μ*m/mL dexamethasone, and 1 *μ*g/mL of insulin). Two days after stimulation with MDI (day 2), the medium was changed to an insulin medium (DMEM containing 10% fetal bovine serum and 1 *μ*g/mL of insulin). Two days later (day 4), the medium was changed to 10% FBS/DMEM. The cells were fed with 10% FBS/DMEM every 2 days. Full differentiation was achieved by day 8.

### 2.3. Oil Red O Staining

To determine both adipogenic potential and fat accumulation, we stained the cells with oil red O (Sigma Chemical Co., St. Louis, MO). On day 8, the cultured cells were washed thrice with phosphate-buffered saline (PBS) and then fixed with 4% formaldehyde for 30 min at room temperature. The cells were washed 3 times more with PBS and stained with 0.5 *μ*g/mL oil red O for 15 min. After rinsing with PBS, we captured representative photomicrographs using a microscope and measured fat accumulation.

### 2.4. AdipoRed Assay

3T3-L1 cells were cultured in 6-well plates at a density of 3 × 10^5^ cells per well and set up to differentiate in the presence and absence of AG. On day 2 and day 8 of differentiation, the cells were exposed with AdipoRed (Lonza, Houston, TX, USA), and intracellular triglyceride (TG) level was analyzed.

### 2.5. Leptin Assay

For the periods indicated, 3T3-L1 cells were treated with AG or not. Conditioned media from the final 24 h of culture were collected. Leptin concentrations were determined using an enzyme-linked immunosorbent assay (ELISA; Mouse Leptin Assay Kit, Immuno-Biological Laboratories Co., Tokyo, Japan).

### 2.6. Real-Time PCR Analysis

Total RNA was isolated from the differentiated 3T3-L1 cells and epididymal adipose tissue by using Easy-Blue Reagent (Intron Biotechnology Inc., Gyoenggi-do, Republic of Korea) according to the manufacturer's instructions. Then, total RNA qualification was performed with an Epoch micro-volume spectrophotometer system (BioTek Instruments Inc., Winooski, VT, USA). An equal amount of total RNA was used to synthesize cDNA with a high-capacity cDNA reverse transcription kit (Applied Biosystems, Foster City, CA, USA). The program was set for 10 min of initiation at 25°C, followed by 90 min of incubation at 50°C and 5 min at 85°C. Primers were obtained from Bioneer (Daejeon, Republic of Korea). All primer sequences and annealing temperatures are shown in Table 1. A Step One Plus Real-time PCR system (Applied Biosystems, Foster City, CA, USA) with an SYBR Green Master Mix (Applied Biosystems, Warrington, UK) and primers (Bioneer, Seoul, Republic of Korea) was used to carry out a real-time PCR. The steps were as follows: 10 min at 95°C, 40 cycles of 5 s at 95°C, 45 s at 60°C, a final melting curve of 15 s at 95°C, 1 min at 60°C, 15 s at 95°C for tissues, or for cells, and 5 min followed by 35 repetitive thermal cycles (94°C for 30 s, 60°C for 30 s, and 72°C for 30 s). Fold changes of gene expression were calculated using the comparative threshold cycle (Ct) method (Applied Biosystems). The data were normalized for the initial control, GAPDH.

### 2.7. Western Blot Analysis

The 3T3-L1 cells were lysed in a lysis buffer (25 mM HEPES, pH 7.4, 100 mM NaCl, 1 mM EDTA, 5 mM MgCl_2_, 0.1 mM dithiothreitol, and protease inhibitor mixture). Determination of the protein was carried out on Tris-glycine SDS-polyacrylamide gels, followed by a transfer to PVDF membranes (EMD Millipore Corporation, Billerica, MA, USA). Then, the membranes were blocked with 5% skim milk in Tris-buffered saline containing 0.1% tween 20 (TBST) at room temperature for 1 h and incubated at 4°C overnight with 1 : 1000 dilutions of primary antibodies. The following day, the membranes were washed with TBST 3 times for 10 min each and made to react with 1 : 2500 dilutions of horseradish peroxidase-conjugated secondary antibody for 2 h at room temperature. After the reaction, the complexes were visualized with ECL plus detection reagents (GenDEPOT, Barker, TX, USA).

### 2.8. Animal Experiments

Male C57BL/6J mice, weighing 15–17 g at an age of 3-4 weeks, were purchased from Daehan Biolink (DaeJeon, Republic of Korea). They were housed (5 mice/cage) under a 12 h light-dark cycle, at 23 ± 2°C, and a relative humidity of 55% ± 10%, under conditions that followed the NIH Guide for the Care and Use of Laboratory Animals; they were allowed diet and water *ad libitum*. After 1 week of acclimation, 40 mice were divided randomly and equally into 4 groups of 10 each. Mice in group 1 (CON) were fed standard chow and those in group 2 (HFD) a HFD (40% fat). group 3 (AG1) consisted of mice fed with the same HFD, which included 1% AG extract (1% w/w in HFD), and group 4 (AG5) consisted of mice fed HFD with 5% added AG extract (5% w/w in HFD). Mice were fed experimental diet for 10 weeks. Body weight was recorded weekly. After an overnight fast at the end of the experimental period, tissue collection was performed. The animals were sacrificed by cervical spine dislocation and the livers, epididymal, retroperitoneal and total fat pads were dissected, weighed immediately, and frozen at −80°C.

### 2.9. Histological Analysis

The liver and the epididymal adipose tissues were fixed with 4% paraformaldehyde and embedded in paraffin. Sections of 4 and 8 *μ*m were cut and stained with hematoxylin and eosin (H&E) for analysis of adipocyte diameter, surface area, and number. The stained slides were observed with a SZX10 microscope (Olympus, Seoul, Republic of Korea) and photographed.

### 2.10. HPLC Analysis

The HPLC apparatus was a Gilson System equipped with a 234 autosampler, a UV/VIS-155 detector, and a 321 HPLC Pump (Gilson, Seoul, Republic of Korea). A Luna 4.60 × 250 mm C18 reversed-phase column with 5 *μ*m particles (Phenomenex, CA, USA) was used. The column eluent was monitored at UV 246 nm, following which all solvents were degassed with a micromembrane filter (PTFE, Advantec, Tokyo, Japan). Chromatography was performed at room temperature at a flow rate of 0.5 mL/min, and 10 *μ*L was analyzed for 50 min. In brief, seven CQs and the four extracts dissolved in 80% MeOH were filtered using a syringe filter and injected for HPLC analysis. The two mobile phases of 0.05% phosphoric acid (solvent A) and MeOH (solvent B) were used for gradient elution at the rate of 1.00 mL/min: 0–10 min, 60% A : 40% B; 10–20 min, 50% A : 50% B; 20–30 min, 40% A : 60% B; 30–35 min, 60% A : 40% B. The two flavonoids, astragalin and kaempferol, were also used as the standard compounds for the analysis. Under these HPLC identical conditions, the retention times of astragalin and kaempferol were 14.4 min and 24.3 min, respectively. Plotting the peak area (*y*, counts) versus concentration (*x*, *μ*g/mL), regression equations of *y* = 27.10*x* + 50.50 (*R* ^2^ = 0.998) and *y* = 54.85*x* + 287.01 (*R* ^2^ = 0.999) for astragalin and kaempferol, respectively. Sample solutions were injected into the HPLC system at 1.000 mg/mL, and the contents were determined from the regression equation.

### 2.11. Statistical Analysis

The results are presented as the mean ± S.E.M. of the 3 independent experiments. The data were analyzed by one-way analysis of variance (ANOVA); statistical analyses were performed using SPSS (Version 19.0). A *P* value of <0.05 was considered statistically significant.

## 3. Results

### 3.1. AG-Mediated Inhibition of Adipogenesis in Preadipocytes

**Figure 1:**
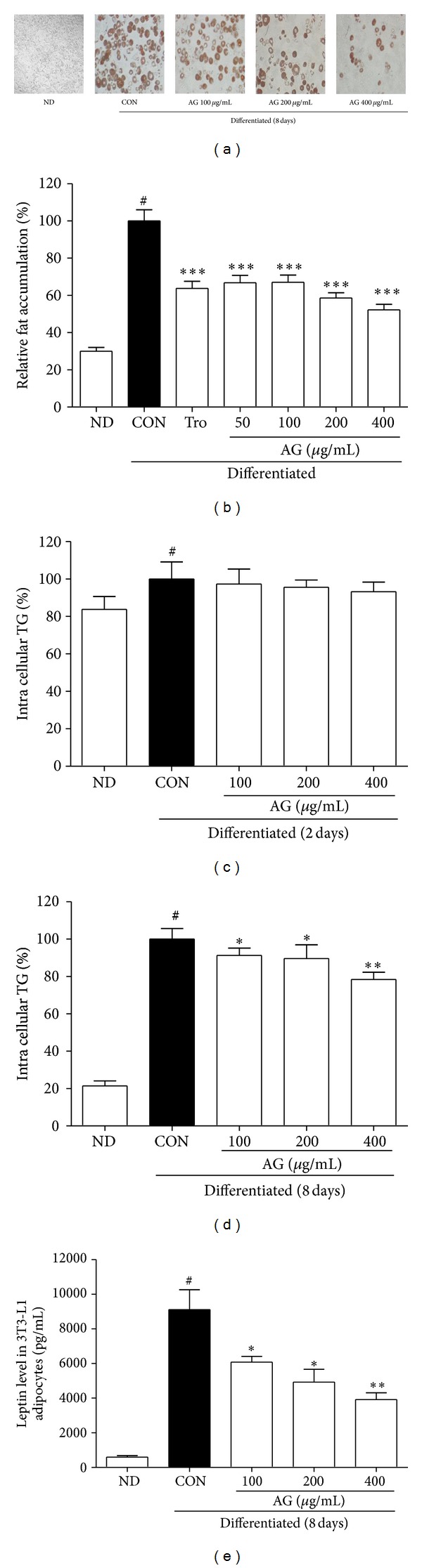
Effects of AG on adipocyte differentiation. (a) Fat droplets in adipocyte differentiated for 8 days with or without AG were stained with oil red dye. (b) Relative lipid accumulation was calculated. Troglitazone was used as a positive control. Accumulation of intracellular triglycerides was calculated by AdipoRed assay at (c) day 2 and (d) day 8 in differentiated adipocyte. (e) Leptin levels in adipocyte with or without AG. ^#^ *P* < 0.05 versus CON group. *P < 0.05 versus HF group, **P < 0.01 versus HF group, and ***P < 0.001 versus HF group.

To determine whether AG has an antiobesity effect, we carried out adipocyte differentiation using 3T3-L1 cells, both in the presence and the absence of AG. After 8 days, oil red O was added to the cultured cells, and fat accumulation and intracellular triglyceride levels were analyzed. Troglitazone, an antidiabetic and anti-inflammatory drug [16], was used as a positive control for the inhibition of fat accumulation. As shown in Figure 1, the number and size of cytosolic lipid droplets markedly decreased (Figure 1(a)), and reduction in fat accumulation, dependent on concentration, was observed (Figure 1(b)). Intracellular TG was analyzed with the AdipoRed assay. On day 2, the accumulation of TG in the differentiated cells was similar to that in the AG-treated cells (Figure 1(c)). After 8 days, the AG-treated differentiated cells showed significantly lower TG levels, exhibiting a dose-dependent change, than those in the untreated differentiated cells (Figure 1(d)). To assess cellular capacity to produce adipocyte-derived hormones, we monitored the expression of leptin, a well-documented hormone with anti-diabetic properties, in cells of adipocyte lineage. Significant suppression of leptin protein secretion was observed in 3T3-L1 cells by the treatment with AG (Figure 1(e)).

### 3.2. AG Regulation of the Expression of Adipogenic Genes in Adipocytes

**Figure 2:**
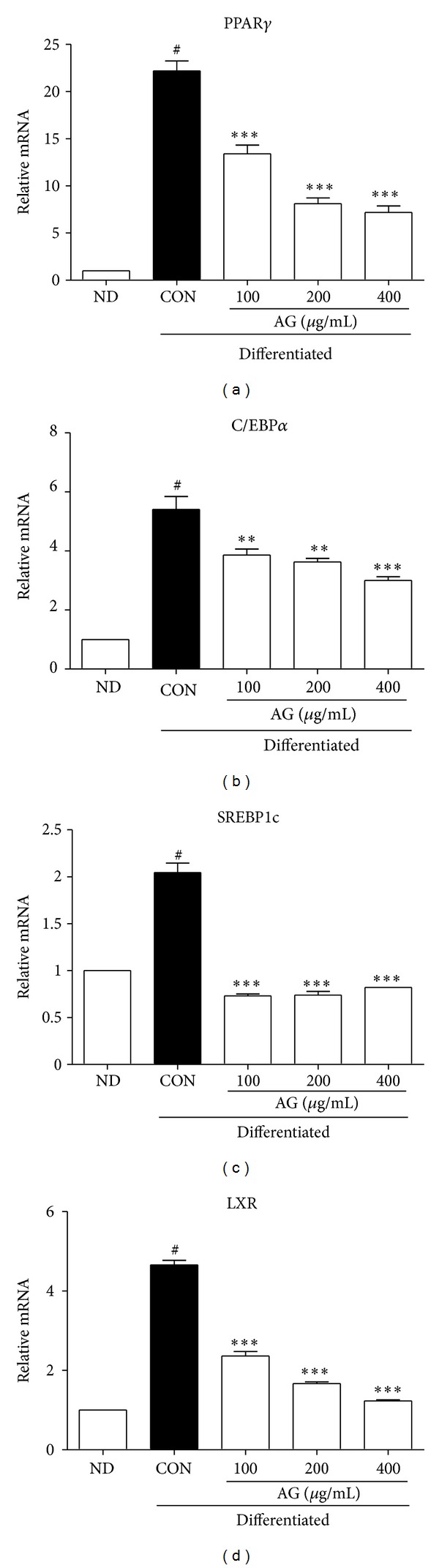
Effects of AG on expression of adipogenic markers. Differentiated adipocytes were treated with 100, 200, and 400 *μ*g/mL respectively. Cells treated with AG were harvested at day 8, and the expressions of genes were analyzed by real-time PCR. Each bar represents the mean ± SEM of triplicate PCR reactions, and mRNA samples were obtained from experimental adipocyte cells. ^#^ *P* < 0.05 versus ND group. *P < 0.05 versus CON group, **P < 0.01 versus CON group, and ***P < 0.001 versus CON group.

The differentiation of adipocytes from preadipocytes is correlated with the expression of adipogenic genes. PPAR*γ*, C/EBP*α*, and SREBP1c are key regulators of adipogenesis, and LXR is also involved. After cell differentiation for 8 days in the presence or absence of 100, 200, and 400 *μ*g/mL of AG, the expression levels of adipogenic markers were determined by real-time PCR. The 8-day AG treatment of cells reduced the mRNA expression of PPAR*γ*, C/EBP*α*, SREBP1c, and LXR (Figure 2). 

### 3.3. AG-Mediated Reduction of Body Weight, Visceral Epididymal, Retroperitoneal, and Total Fat-Pad Weights, and Liver Weight in HFD-Induced Obese Mice

**Figure 3:**
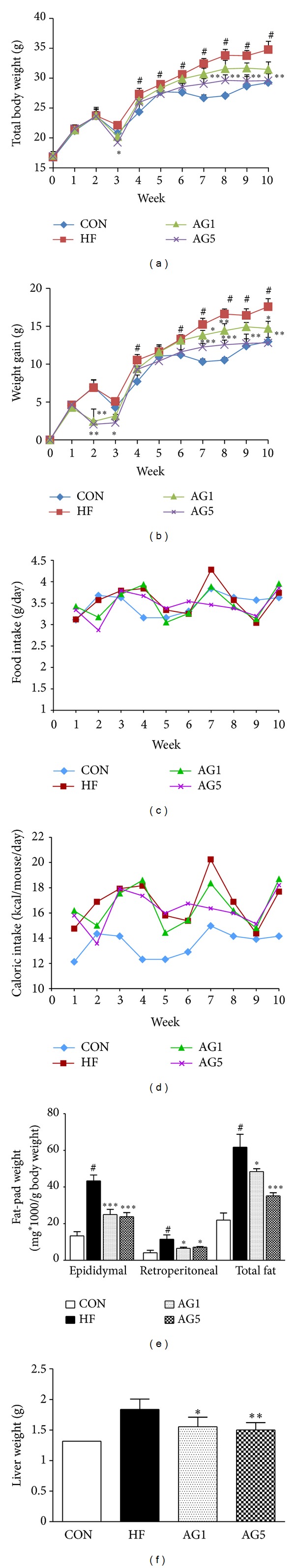
Effects of AG on body weight gain, fat-pads weights, and liver weights of N, HF, AG1, and AG5 group mice. Mice were fed normal diet or HFD for 10 weeks in the presence or absence of AG. (a) Changes in total body (g), (b) changes in body weight gain (g), (c) food intake, (d) caloric food intake, (e) visceral fat-pad weight, and (f) liver weights in each group are shown. ^#^ *P* < 0.05 versus CON group. *P < 0.05 versus HF group, **P < 0.01 versus HF group, and ***P < 0.001 versus HF group.

Figure 3 indicates the changes in total body, as well as epididymal, retroperitoneal, and total fat pads and liver weight, all of which were significantly greater in the HFD group than in the control group. The increases in body weight, fat weights, and liver weight in the AG1 group were lesser than those in the HFD group (Figures 3(a), 3(b), 3(e), and 3(f)). The corresponding weight increases in the AG5 group were significantly lesser than those in the HFD and CON groups (Figures 3(a) and 3(b)). The food intake amount was not different among each group (Figure 3(c)), so the effect of AG is not an impact of reduced food intake. However, the HFD groups showed higher caloric food intake (kcal/day/mouse) than the CON group (Figure 3(d)). The caloric food intake was also not different among each groups.

### 3.4. AG-Mediated Amelioration of HFD-Induced Fat Accumulation in Liver and Adipose Tissue

**Figure 4:**
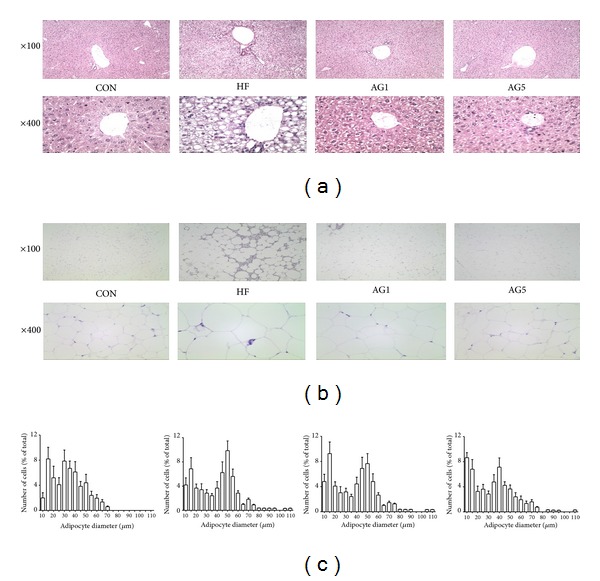
Effects of AG on fat accumulation. Representative histological images of the (a) liver, (b) epididymal adipose tissue as assessed by H&E staining and examined using a light microscope; magnification: ×100, ×400. (c) Adipocyte number and diameter.

To determine whether or not the decreased body, visceral, and liver weights were due to reduced accumulation of fat, we stained representative liver and adipose tissues with H&E. As shown in Figure 4, liver sections of the HFD group, unlike those of the CON group, exhibited lipid deposition in the hepatocytes, particularly around the central vein. Considering mild intracytoplasmic hepatocyte vacuolation, the histological liver images of the AG1 and AG5 groups indicate that AG ameliorated the accumulation of lipids (Figure 4(a)). Because fatty degeneration in adipose tissues is easy to identify, we compared the diameter and number of lipid droplets in the adipose tissues. As shown in Figure 4(b), AG1 and AG5 decreased the diameter and the number of lipid droplets markedly (Figure 4(b)).

### 3.5. In Vivo AG-Mediated Modulation of Adipogenic-Related Signaling Molecules in Adipose Tissue

**Figure 5:**
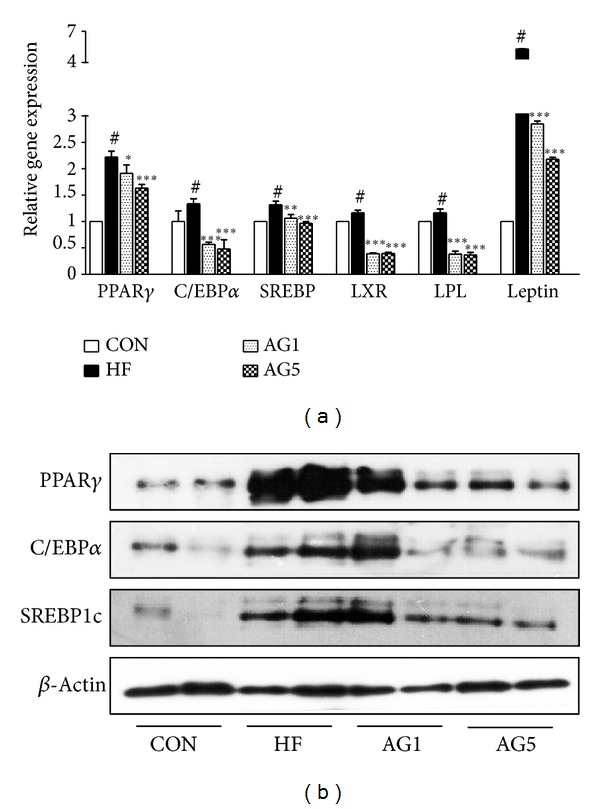
Effects of AG on expression of adipogenic signal genes. (a) Real-time PCR analysis of epididymal adipose tissue from experimental mice shows the expression of adipogenesis-related transcription. Data were normalized to the GAPDH mRNA levels and then compared to CON group measurements, which were assigned a value of 1.0. (b) Protein levels of PPAR*γ*, C/EBP*α*, and SREBP1c were determined by Western blotting. Each bar represents the mean ± SEM of 3 independent experiments of the RNA and protein samples obtained from 5 mice per each group. ^#^ *P* < 0.05 versus CON group. *P < 0.05 versus HF group, **P < 0.01 versus HF group, and ***P < 0.001 versus HF group.

To analyze the inhibitory effects of AG on the adipogenic mRNA expression induced by HFD in epididymal white adipose tissue, we conducted a quantitative real-time PCR analysis. As it affected adipogenesis in vitro, AG attenuated the expression of mRNA related to adipogenesis. The expression of PPAR*γ* was evidently reduced in the epididymal tissue taken from AG-treated HFD mice. The expression of C/EBP*α*, a downstream target of PPAR*γ*, also decreased significantly. Other adipogenic markers such as SREBP1c, LXR, LPL, and leptin were reduced markedly in the epididymal tissue of AG-treated mice (Figure 5(a)). In parallel with mRNA expressions, smaller amounts of PPAR*γ*, C/EBP*α*, and SREBP1c proteins were produced in epididymal adipose tissue obtained from AG-treated HFD mice than from mice given HFD alone (Figure 5(b)).

### 3.6. Characterization of AG Using HPLC Systems

**Figure 6:**
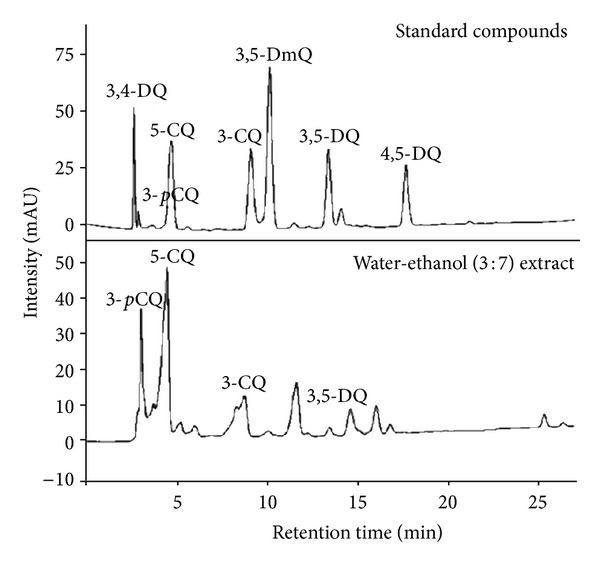
HPLC chromatograms of the extract from AG.

The CQs were analyzed by HPLC using seven standard compounds. The CQs identified by HPLC using standard compounds were 3-pCQ, 3,4-DQ, 5-CQ, and 3,5-DQ (Figure 6). When the contents of the two flavonoids were quantitatively compared, astragalin and kaempferol were 13.2 mg/g and 0.23 mg/g (Table 2).

## 4. Discussion

The number of people with obesity and obesity-related diseases, such as diabetes mellitus, hypertension, coronary artery disease, and cancers [17], has increased at an alarming rate all over the world [18]. Consequently, the idea of developing antiobesity drugs with no undesirable side effect has become a hot topic [19]. Herbal medicine has been looked at as a complementary treatment [20]. To keep pace with global research trends, we investigated the antiobesity effects of AG, a plant found in mountainous terrain. The results of this study indicated the antiadipogenic effects of AG, both in vitro and in vivo. In vitro, the AG blocked adipocyte differentiation, as well as the accumulation of fat and intracellular TG in 3T3-L1 adipocytes. AG also decreased leptin levels dose-dependently, which are a marker of adipocyte differentiation. In vivo, AG reduced body weight, fat-pad weight, and liver weight. In addition, AG decreased the expression of adipogenic signal genes in experimental obese mice.

Several reports have indicated PPAR*γ*, C/EBP*α*, and SREBP1c as the major transcriptional genes involved in adipogenesis [3, 21, 22]. PPAR*γ* is a ligand-activated transcription factor that plays an important role in the regulation of obesity. It exists as a heterodimer with another nuclear hormone receptor, retinoid X receptor, or RXR to bind to DNA, and it is actively involved in adipogenesis [23]. Adipocyte differentiation provokes PPAR*γ* activity, and it transfers hormonal stimulation to its target genes such as C/EBP*α* and LPL [19, 24]. Although C/EBP*α* is induced by PPAR*γ*, C/EBPβ, and C/EBP*δ*, members of C/EBP family are detected at a very early stage of adipocyte differentiation, followed by PPAR*γ*, and they accelerate the process of adipogenesis, acting with PPAR*γ* [25]. Despite its role as a target gene of PPAR*γ*, C/EBP*α* acts in a positive-feedback loop to express PPAR*γ* and other adipogenic-related genes. It maintains an enhanced differentiated state [26]. SREBP1c also has a profound effect on adipogenesis; it stimulates the expression of many lipogenic genes and regulates fatty acid and glucose metabolism [27]. Furthermore, it has been suggested that SREBP1c is correlated with the production of an endogenous PPAR*γ* ligand that reinforces PPAR*γ* activity and, for this reason, PPAR*γ* is a target gene of SREBP1c [28, 29]. Considering the significance of these factors that take a leading position in adipogenesis, AG seems to suppress adipocyte differentiation by blocking PPAR*γ*, C/EBP*α*, and SREBP1c activation.

LXR is known to regulate cholesterol and fatty acid metabolism in liver tissue and in macrophages [30]. Recently, LXR—considered one of the major genes for regulation of lipid metabolism—has also been found to be involved in the process of adipocyte differentiation. It affects the adipocyte-specific gene expression and adipogenesis. LXR activation meditates lipogenesis and adipogenesis by inducing SREBP1c and PPAR*γ* [7]. Because PPAR*γ* directly regulates LXR*α*, which is one of the LXR families, PPAR*γ* and LXR form a positive-feedback relation in adipocyte differentiation [31]. In this study, AG decreased LXR levels in 3T3-L1 cells and in epididymal adipose tissues.

Leptin, a hormone secreted largely by adipose tissue to maintain body fat, regulates food intake and energy expenditure through its action on the hypothalamus. When the level of body fat mass decreases, plasma leptin levels fall, stimulating appetite and suppressing energy expenditure until fat mass is restored. Conversely, when fat mass increases, levels of leptin increase and suppress appetite until weight is lost. Thus adipose tissue mass seems to be under the control of leptin [32]. In brief, an activated leptin gene links to a state of obesity. In our results, the expression of the leptin gene was decreased by AG, apparently signifying that AG attenuates weight gain and fat accumulation by inhibiting leptin release. Leptin resistance, the hallmark of high-Fat diet induced obesity model, is a phenomenon in which circulating leptin levels decrease in response to target tissues, such as adipose tissue, muscles, liver, and hypothalamus. In most cases, the leptin resistance could be either primary or acquired. In human adiposity, although there exit substantial individual differences at some specific levels of body fat, leptin concentrations in the circulation are associated with adipose tissue [33]. Therefore, the close correlation between hyperleptinemia and body weight has resulted in recognition of leptin resistance as a cause of obesity. In our present study, leptin resistance is developed by the administration of a high-fat diet. Subsequently, treatment with AG decreased leptin gene level, and this means that AG may ameliorate leptin resistance.

In our experiment, we used a small amount of the AG extract at doses of 1% and 5% AG (w/w in HFD). We usually use 10–20% doses for evaluating the antiobesity effect in animal models [34]. However, our previous data showed that AG has a good lipid lowering effect in lower drug doses [15]. Thus, in this study we reduced AG doses (1% and 5%). AG is able to effective in spite of the low doses (1% and 5%). Thus, mice were fed HFD mixed with AG extract, and the method of feeding mice meant that their intake of AG was not equal among them. If they were given larger doses of AG extract or if the AG ingestion modality was changed to oral administration or intraperitoneal injection, an antiobesity effect could be more evident.

Adipose tissue is considered a crucial factor in the regulation of many pathological processes, and it is linked to both the immune and the metabolic systems. For that reason, obesity is associated with both a chronic inflammatory response and immune functions [35]. Further studies are needed to identify the effects of AG on inflammation and on the immune system.

Caffeoylquinic acids (CQs) are esters of caffeic and quinic acids and are phenolic compounds. They exist naturally as chlorogenic acid (3-CQ), cryptochlorogenic acid (4-CQ), neochlorogenic acid (5-CQ), cynarin (1,5-diCQ), isochlorogenic acid A (3,5-diCQ), isochlorogenic acid B (3,4-diCQ), and isochlorogenic acid C (4,5-diCQ) [36]. Their various biological functions include antioxidant, antiinflammatory, anti-microbial, anti-skin-aging, antihypercholesterolemia, and antihyperglycemia activities. Previously, we reported that AG had high quantity of CQs [14]. In this study, we used CQ-rich extract from AG, and downregulation of leptin gene could be related with leptin sensitivity by CQ compound. This effect can be induced by reduction of triglyceride and insulin levels.

## 5. Conclusion

We have demonstrated that AG exerts antiobesity effects both in vitro and in vivo. Its effects on obesity are based on the downregulation of adipogenic-related transcription factors, suggesting that AG has a potential as a therapeutic agent for obesity and obesity-related diseases.

## Figures and Tables

**Table 1:** Primer sequences and PCR conditions.

Gene name	*T*_*m*_ (°C)	Size (bp)	Sequence 5′-3′
Peroxisome proliferator-activated receptor gamma (PPAR*γ*)	55	176	F: TCGGAATCAGCTCTGTGGA R: CCATTGGGTCAGCTCTTGTG
CCAAT/enhancer binding protein alpha (C/EBP*α*)	55	151	F: ACAACGCAACGTGGAGAC R: ACCAAGGAGCTCTCAGGCAG
Sterol regulatory element binding transcription factor 1 (SREBP1c)	55	198	F: ATCGCAAACAAGCTGACCTG R: AGATCCAGGTTTGAGGTGGG
Liver X receptor (LXR)	55	119	F: TCCTACACGAGGATCAAGCG R: AGTCGCAATGCAAAGACCTG
Lipoprotein lipase (LPL)	55	158	F: AGGACCCCTGAAGACACAGC R: TTGGGCACCCAACTCTCATA
Leptin	55	143	F: CTCCAAGGTTGTCCAGGGTT R: AAAACTCCCCACAGAATGGG

**Table 2:** Content of caffeoylquinic acids and flavonoids in AG.

Compounds	Contents (mg/g)
3,5-DQ	4.43
5-CQ	13.69
3-CQ	3.25
3-pCQ	34.06
Astragalin	13.2
Kaempferol	0.23
