# Corrigendum to “Antiadipogenic Effects of* Aster glehni* Extract: In Vivo and In Vitro Effects”

**DOI:** 10.1155/2019/2459192

**Published:** 2019-01-10

**Authors:** Heon-Myeong Lee, Gabsik Yang, Tae-Gue Ahn, Myung-Dong Kim, Agung Nugroho, Hee-Juhn Park, Kyung-Tae Lee, Wansu Park, Hyo-Jin An

**Affiliations:** ^1^Department of Pharmacology, College of Oriental Medicine, Sangji University, Wonju-si, Gangwon-do 220-702, Republic of Korea; ^2^Department of Physiology, College of Oriental Medicine, Sangji University, Wonju-si, Gangwon-do 220-702, Republic of Korea; ^3^Department of Pharmaceutical Engineering, Sangji University, Wonju-si, Gangwon-do 220-702, Republic of Korea; ^4^Department of Pharmaceutical Biochemistry, College of Pharmacy, Kyung Hee University, Seoul 130-701, Republic of Korea; ^5^College of Oriental Medicine, Kyungwon University, Seongnam 461-701, Republic of Korea

In the article titled “Antiadipogenic Effects of* Aster glehni* Extract: In Vivo and In Vitro Effects” [1], the name of the first author was given incorrectly as Heon-Myung Lee. The author's name should have been written as Heon-Myeong Lee. The revised authors' list is shown above. Additionally, the Authors' Contribution section should be corrected as follows: Heon-Myeong Lee and Gabsik Yang contributed equally to this work.
